# Correlation of Medical College Admission Test Scores and Self-assessment Materials with the United States Medical Licensing Examination Step 1 Performance

**DOI:** 10.7759/cureus.7519

**Published:** 2020-04-02

**Authors:** Zachary A Seal, Wouter Koek, Ramaswamy Sharma

**Affiliations:** 1 Medical Education and Simulation, University of Texas Health Science Center at San Antonio, San Antonio, USA; 2 Psychiatry, University of Texas Health Science Center at San Antonio, San Antonio, USA; 3 Cell Systems and Anatomy, University of Texas Health Science Center at San Antonio, San Antonio, USA

**Keywords:** usmle step 1, mcat, uworld, nbme, score predictor, question bank, residency

## Abstract

Purpose

Candidates' performance on the United States Medical Licensing Examination (USMLE) Step 1 examination had been correlated with the Medical College Admission Test (MCAT). However, in 2015, a new MCAT format was released and its correlation with Step 1 remains to be fully analyzed. Preparation for Step 1 typically involves purchasing and perusing practice tests from the National Board of Medical Examiners (NBME) and UWorld; however, their predictive value to performance on Step 1 remains to be ascertained, especially with the release of five new NBME practice tests. Additionally, there is a need for accurately predicting Step 1 scores to self-evaluate study progress and reduce student anxiety.

Rationale

Program directors rank USMLE Step 1 scores as the number one criterion in selecting interviewees for residency. Step 1 scores are more important than Step 2 scores, Dean’s letter, or other letters of recommendation in determining the overall ranking of a candidate after interviews.

Hypotheses

The authors hypothesized that the new MCAT scores correlated positively with Step 1 scores and that the new NBME practice tests were more predictive of performance on Step 1 as compared to old NBME tests.

Methods

Linear regression analysis followed by either analysis of variance (ANOVA) or Student's t-tests were used to analyze 399 responses. Data obtained was used to update an existing Step 1 score predictor, which was then validated.

Results

A positive correlation between the MCAT (average score: 510.1 ± 6.3) and Step 1 scores (average score: 246.1 ± 14.2) was observed. While new NBME practice tests were more predictive of Step 1 scores than old NBME tests, UWorld test scores were the most predictive. Students who practiced with the new NBME practice tests scored significantly higher than students who did not use them. However, students using any of the UWorld practice tests did significantly better than students who practiced using only NBME practice tests but not UWorld practice tests. Ironically, NBME16,the second-most correlativetest to Step 1 performance, is no longer available for purchase. Overall, taking six or more practice tests significantly enhanced Step 1 scores; the optimal number of tests was found to be between six and nine. The predicted score by an updated Step 1 score predictor was within 3.8 points or 1.6% of the actual Step 1 score.

Conclusions

We believe this study will aid in the selection and purchase of appropriate self-assessment tests as preparatory material for the USMLE Step 1 examination. It will also introduce them to an existing Step 1 score predictor that will help determine their readiness for Step 1.

## Introduction

The United States Medical Licensing Examination (USMLE), sponsored by the Federation of State Medical Boards (FSMB) and the National Board of Medical Examiners (NBME), consists of three examinations or “steps” that assess medical student and resident competency for practicing safe medicine in the United States [1]. Step 1 tests basic science concepts while Step 2 evaluates clinical knowledge [2]. Step 3 is taken during the first year of residency and evaluates the application of medical knowledge to the supervised practice of medicine. Of these, Step 1 scores have been shown to play an important role in residency selection by standardizing academic achievements of students from different schools, in predicting success in clinical clerkships and Step 2 examinations, and in board examinations taken during residencies [3-9]. Therefore, parameters influencing student performance on Step 1 examinations such as the Medical College Admission Test (MCAT) and related preparatory materials are of interest to both students as well as medical schools.

The MCAT is a standardized examination that forms an integral part of the application process to medical schools across the United States. Along with undergraduate grade point average (GPA) and extracurricular activities such as shadowing physicians, recommendation letters, and a personal statement, the MCAT is a critical component of medical school admissions [10]. The MCAT not only provides the admissions committees with an objective leveling metric for evaluating candidates from different schools with variable GPAs but has also been shown to positively correlate with performance on USMLE Step 1 [11-13]. However, the format and scoring of the MCAT were modified extensively in April 2015 from a three-section test with a maximum possible score of 45 to a four-section test with a maximum possible score of 528. The fourth section in the 2015 edition focused on psychology and sociology. While scores on the new MCAT format have been recently correlated with performance in the first year of medical school, only one recent study, at the University of Minnesota Medical School-Twin Cities, has correlated the scores on the new MCAT format with Step 1 scores [14,15]. 

Another important factor influencing Step 1 scores is the use of specific study and evaluation materials while preparing for the examination, such as question banks. Indeed, the use of question banks has a positive effect on Step 1 scores, especially for those with lower MCAT scores [16-19]. Similarly, performance on self-assessment tests may also help predict Step 1 scores [18]. These practice tests are provided by NBME, known as Comprehensive Basic Science Self-Assessment (CBSSA), as well as other companies such as UWorld. While previous studies have established a positive correlation between NBME CBSSA scores and Step 1 scores, NBME discontinued several of these tests in spring 2019 and introduced five new practice examinations [18]. Specifically, five old practice examinations were retired (Forms 13, 15, 16, 17, and 19) and were replaced by five new practice examinations (Forms 20-24); the correlation of these new tests with Step 1 performance is not yet known. Similarly, UWorld also provides students with question banks such as QBank and practice tests such as UWorld Self Assessment 1 (UWSA1) and UWorld Self Assessment 2 (UWSA2), which are widely used. Correlations of these self-assessment tests with Step 1 scores will help identify specific tests that can closely predict performance on Step 1 examinations and allow students to gauge their progress during their preparations. Since there is a cost associated with each test, it also becomes important to determine the minimum number of practice tests required to optimize Step 1 performance.

Given the importance of Step 1 in matching residency and the consequent student anxiety, attempts have been made to create score predictors that use the correlation between MCAT and question banks with Step 1 scores [20]. For example, a model was developed using scores on the Comprehensive Basic Science Examination (CBSE; a scored test administered by NBME and taken by medical students to prepare for Step 1), UWorld QBank, first-year grades, and financial need [17]. This model was able to explain 62.3% of the variance in Step 1 scores and was a good first step; however, this model required values for each of these variables for prediction such that students at a school that did not offer CBSE or provided “Pass” or “Fail” grades instead of letter grades would be unable to benefit. Interestingly, a score predictor was posted on the web forum, Reddit (https://www.reddit.com), that appeared to closely predict Step 1 scores without requiring a student to enter every variable. This score predictor took into account practice examination scores from NBME and UWorld, in addition to question bank scores from Kaplan, UWorld, and USMLE-Rx. However, the score predictor required the addition of the new NBME tests and remained to be validated.

In this study, we examined the correlation of Step 1 scores with the new MCAT format, question banks, and self-assessment tests; we also updated and validated an existing Step 1 score predictor, and identified the minimum number of tests required as well as an optimal study period to facilitate success in the Step 1 examinations.

## Materials and methods

Data collection and analysis

We posted an anonymous survey on Reddit requesting users who had already taken the USMLE Step 1 examination to submit their scores along with all relevant practice test scores as well as their MCAT score and the dates when the tests were taken. All accrued data were downloaded and compiled into a single Excel sheet. A total of 466 responses were received. Reddit usernames were used to determine duplicity of information; duplicate values were found and deleted. This was followed by deleting responses that only listed the USMLE Step 1 scores but not practice test scores such that a total of 399 scores could be used. However, the number of responses for each test varied based on the number of students using that particular test. Data were then de-identified by removing Reddit usernames and used for statistical analysis.

Statistics

Linear regression analysis was used to determine the correlation between scores on the new MCAT format and Step 1 score as well as between the old MCAT format and Step 1 score received by the student. Similarly, linear regression analysis was again used to determine the correlation between each of the selected practice tests and question banks with the Step 1 score. Multiple regression analysis could not be performed since students differed in the number and type of practice tests and questions banks; therefore, p-values for practice tests or question banks were adjusted using the Holm-Sidak test. One-way analysis of variance (ANOVA) or Student’s t-tests were used to determine significance using GraphPad Prism version 8.03 (GraphPad Software, San Diego, CA) as appropriate. The Shapiro-Wilk test was used to determine normality. A p-value of <0.05 was considered statistically significant.

Step 1 score predictor

A Step 1 score predictor that utilizes performance on practice tests and on question banks to predict the USMLE Step 1 scores has been available on Reddit for the past several years (https://drive.google.com/file/d/1r2ir9uEU58PzKHVfYFaHhZG6mKs_YTNI/view). The calculator utilizes the lines of best fit for each practice test to give an estimated score. The adjusted R^2^ value of the line of best fit for each practice test is then used to weight the estimated scores used in the calculation. The average of all such weighted estimated scores is used towards predicting the USMLE Step 1 score. In addition to the scores themselves, the date of the practice tests is also included in the calculations such that practice tests that are taken closer to the actual USMLE examination are weighted more heavily than those taken much earlier. However, with the introduction of the new NBME practice tests (NBME Forms 20-24), it was necessary to modify the calculator (https://drive.google.com/file/d/1Ry-BCrt8BT-cVsrXP2Yu6el-KrAeP6rX/view?usp=sharing). The modified calculator was tested using 19 scores that were not part of the original 466 responses.

## Results

The positive correlation of MCAT scores with USMLE Step 1 scores

**Figure 1:**
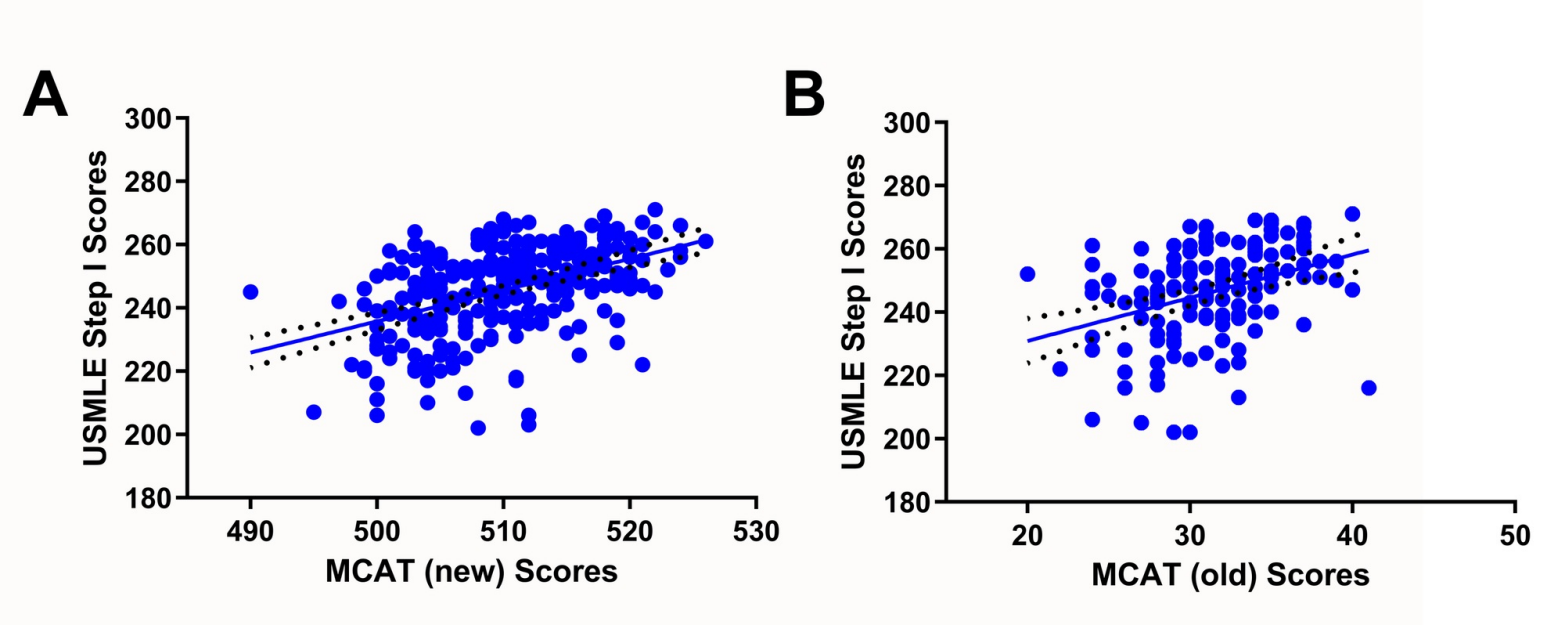
Correlation of MCAT scores with USMLE Step 1 scores A: linear regression analysis of scores obtained on the new format of the MCAT and USMLE Step 1 scores (R^2 ^= 0.208; slope = 0.9873 ± 0.116; 95% CI = 0.761-1.214; p: 8.301e-16; n = 281); B: linear regression analysis of scores obtained on the old format of the MCAT and USMLE Step 1 scores (R^2 ^= 0.130; slope = 1.366 ± 0.296; 95% CI = 0.779-1.953; p: 1.11e-05; n = 141) MCAT: Medical College Admission Test; USMLE: United States Medical Licensing Examination

The mean score of survey participants on the new MCAT was 510.1 ± 6.3 (range: 490-526); this score is similar to the average MCAT score of matriculants to United States allopathic medical schools in the year 2017-2018 (510.4 ± 6.6) [21,22]. The mean Step 1 score of survey participants was 246.1 ± 14.2 (range: 202-271); the national average is 231 [22]. The mean score of survey participants on the old MCAT was 31.3 ± 4.0 (range: 490-526). While the old MCAT positively correlated with USMLE Step 1, the new MCAT correlated better with performance on Step 1 (Figure 1).

The association of new NBME CBSSA tests with improved USMLE Step 1 scores

New NBME CBSSA tests (Forms 20-24) were released in Spring 2019 with concurrent removal of practice tests 13, 15, 16, 17 and 19. The contributions of the new NBME tests towards USMLE Step 1 scores have never been assessed. Of students who participated in this survey, those who did not take any of the new NBME practice tests scored 239.8 ± 15.7, and those who took at least one new NBME practice tests scored 247.1 ± 13.7. A two-tailed t-test analysis revealed a t-value of -3.69 [degree of freedom (df) = 397] with a p-value of 0.0003, indicating that students who took at least one of the new NBME practice tests scored better than their counterparts who did not. Interestingly, individuals who took four or all of the new NBME practice tests (mean = 249.6 ± 13.8) scored significantly higher than those who took between one and three examinations (mean = 245.8 ± 13.5) respectively (t-value: -2.48, p: 0.007). A simple regression analysis indicated that there was no significant difference between students taking one or more of the new NBME practice tests (R^2 ^= 0.023; p: 0.099).

To determine if those students who used the new NBME practice tests also used greater numbers of other practice tests, we divided our cohort into six groups of students who took from zero to all five of the new NBME tests. We then determined the average number of other tests (excluding the new NBME tests) taken by these groups. Those who did not use any of the new NBME tests took an average of 3.448 ± 2.429 other tests (n = 58), those who used one of the new NBME tests took an average of 3.804 ± 1.586 (n = 46), those who used two of the new NBME tests took an average of 3.607 ±- 1.276 tests (n = 89), those who used three of the new NBME tests took an average of 3.659 ± 1.492 tests (n = 85), those who used four of the new NBME tests took an average of 3.489 ± 1.098 tests (n = 45), and those who used all five new NBME tests took an average of 4.750 ± 2.198 tests (n = 76). While there was no statistically significant difference between those who took none of the new NBME tests and those who took between one to four of the new NBME tests, there was a significant difference between those took all five of the new NBME tests and those who took none of the new NBME tests (p: 0.0015). These data suggest that while most students did not avail of other additional practice tests, those who took all five of the new NBME tests were also more likely to utilize as many practice tests as available.

The close match between UWSA2 performance and USMLE Step 1 scores

**Figure 2:**
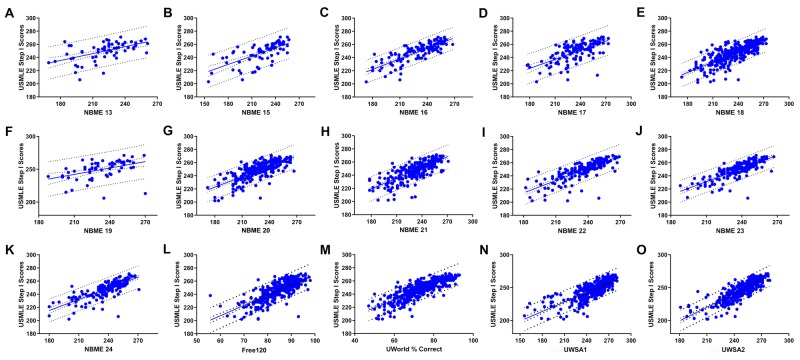
Correlation of practice tests with USMLE Step 1 scores A: NBME 13; B: NBME 15; C: NBME 16; D: NBME 17; E: NBME 18; F: NBME 19; G: NBME 20; H: NBME 21; I: NBME 22; J: NBME 23; K: NBME 24; L: Free 120; M: UWorld QBank; N: UWSA1; O: UWSA2 Lines shown are lines of best fit as well as lines demonstrating 95% confidence limit USMLE: United States Medical Licensing Examination; NBME: National Board of Medical Examiners; UWSA: UWorld Self Assessment

Among all the practice tests analyzed (Figure 2), scores on the UWSA2 exhibited the highest correlation with USMLE Step 1 scores, with an R^2^ value of 0.680 (Table 1). The next-best correlation with Step 1 scores was NBME CBSSA Form 16, with an R^2 ^value of 0.660. UWorld QBank ranked third, with an R^2^ value of 0.656. Since NBME 16 has been discontinued since March 2019, the UWSA2 and QBank now rank at the top. Interestingly, the highest R^2^ value for any of the newer NBME practice tests (20-24) was only 0.620 even though the number of responses for these tests was far greater compared to NBME Form 16.

**Table 1:** Correlation of question banks and self-assessment tests with USMLE Step 1 scores NBME: National Board of Medical Examiners; UWSA: UWorld Self Assessment; SEM: structural equation modeling

Test name	Responses, n	Coefficient of determination, R^2^	Holm-Sidak, adj. p-value	Average score	Slope ± SEM	95% confidence interval of slope
NBME 13	64	0.305	4.454e-006	222.0	0.351 ± 0.067	0.217–0.486
NBME 15	73	0.452	2.164e-010	222.4	0.424 ± 0.055	0.313–0.534
NBME 16	123	0.660	0.000	230.1	0.535 ± 0.035	0.466–0.603
NBME 17	130	0.413	8.882e-016	248.2	0.461 ± 0.047	0.366–0.556
NBME 18	333	0.589	0.000	236.1	0.531 ± 0.024	0.483–0.579
NBME 19	64	0.187	3.549e-004	233.4	0.325 ± 0.086	0.153–0.496
NBME 20	242	0.555	0.000	228.1	0.528 ± 0.031	0.468–0.588
NBME 21	280	0.563	0.000	231.5	0.543 ± 0.029	0.487–0.599
NBME 22	188	0.618	0.000	235.4	0.627 ± 0.036	0.556–0.698
NBME 23	163	0.620	0.000	235.7	0.670 ± 0.041	0.589–0.751
NBME 24	166	0.582	0.000	237.4	0.561 ± 0.037	0.489–0.634
Free 120	351	0.567	0.000	*84.7%	1.589 ± 0.074	1.444–1.735
UWSA 1	360	0.619	0.000	248.1	0.486 ± 0.020	0.446–0.525
UWSA 2	369	0.680	0.000	248.2	0.694 ± 0.025	0.646–0.743
UWorld QBank	383	0.656	0.000	*72.8%	1.191 ± 0.044	1.104–1.277

Additionally, a comparison of the difference between the scores on each practice test with the actual Step 1 scores showed that UWSA2 scores were closest to Step 1 scores (average difference = 6.9 points; Table 2). 

**Table 2:** Difference between scores on practice tests and Step 1 scores *Average difference refers to the average of the differences between each student’s USMLE Step 1 score and their NBME or UWorld practice test NBME: National Board of Medical Examiners; UWSA: UWorld Self Assessment; USMLE: United States Medical Licensing Examination

Test Name	Average practice test score	Average Step 1 score	Average difference*	Responses, n
NBME 13	222.0	250.1	28.6	64
NBME 15	222.4	247.3	25.4	73
NBME 16	230.1	248.8	19.1	123
NBME 17	233.9	248.2	17.1	130
NBME 18	236.1	246.9	12.8	333
NBME 19	233.4	249.7	19.5	64
NBME 20	228.1	248.1	20.7	242
NBME 21	231.5	247.1	16.8	280
NBME 22	235.4	248.7	14.8	188
NBME 23	235.7	248.3	13.8	163
NBME 24	237.4	247.4	11.9	166
UWSA 1	248.1	246.7	10.5	360
UWSA 2	248.2	246.7	6.9	369

To assess the relationship between sample size and the correlation between practice materials and Step 1 scores, a line of best fit correlating the R^2^ values as well as the “n” for each group was created; the R^2^ for that line was 0.245, indicating that while sample size did have a slight effect on correlation, it was not the only factor.

A comparison between the performance of students who took UWorld practice tests and students who did not take a single UWorld practice test

Considering that UWSA2 had the highest single practice test correlation with USMLE Step 1 scores, it was important to determine if students who took either UWSA1 or UWSA2 practice tests or both obtained higher scores in the Step 1 examination as compared to those students who did not use UWorld. A two-tailed t-test indicated a significant difference (t-value: -3.056; p: 0.001) between the mean Step 1 scores of those who took at least one UWorld practice test (246.5 ± 13.9) and those who did not take a single UWorld practice test (236.4 ± 17.2). Similarly, a two-tailed t-test comparing those who took only one UWSA test (242.4 ± 13.2) to those taking both UWSA tests (246.9 ± 13.9) revealed a significant difference (t-value: -1.74; p: 0.042), indicating the importance of UWSA tests in maximizing USMLE Step 1 scores.

To determine if those students who used the UWSA tests took greater numbers of other practice tests, we divided our cohort into three groups: students who took zero, one, or both of the UWSA tests. Students who did not take any of the UWorld tests took 1.632 ± 2.006 other additional tests (n=19); students who took one of the UWorld tests took 3.258 ± 2.236 additional tests (n=31), and students who took both of the UWorld tests took 4.880 ± 2.345 additional tests (n=349). The difference between the groups who took none of the UWorld tests and the groups who took at least one of the UWorld tests was statistically significant (p: 0.013); similarly, there was a statistically significant difference between the groups that took none of the UWorld tests and the groups who took both of the UWorld tests (p: 0.0001). Since the difference between those students who took one UWorld test and those who took both UWorld tests was also statistically significant (p: 0.0002), these data suggest that students who took more UWorld practice tests were also more willing to practice using multiple tests.

The optimal number of tests to maximize USMLE Step 1 scores

This study enabled us to determine the optimal number of practice tests for maximizing USMLE Step 1 score. Because the average number of tests taken by participants in this study was 6.4 ± 2.7 tests, we divided the responses into three categories: those whose used between 0-5 tests (n = 139; mean = 242.5 ± 14.0; median = 246), those who used 6-9 practice tests (n = 217; mean = 247.6 ± 13.7; median = 250) and those who used 10-14 tests (n = 43; mean = 249.6 ± 15.7; median = 253). Because the data within each of the groups were not normally distributed (Shapiro-Wilk normality test), non-parametric Kruskal-Wallis ANOVA was used to compare the medians of the groups (p: 0.000). The Mann-Whitney test indicated a significant difference between those taking zero-five tests and those taking six-nine tests (p: 0.006); however, there was no difference between those taking six-nine tests and those taking 10-14 tests (p; 0.158), suggesting that the optimal number of practice tests was between six and nine. Since the average number of tests taken by participants in this study was six, this may have partly accounted for the relatively high mean USMLE Step 1 score of the participants (246.1 ± 14.2) compared to the national average of 231 in 2018 [22].

Prediction of scores based on performance on practice tests

With the responses collected from the initial survey, we updated an existing Step 1 score predictor found on Reddit. This predictor utilized lines of best fit correlating practice test and question bank scores with Step 1 scores, and weighted the outputs by R^2^ value of each line of best fit as well as by the date taken. Therefore, tests that had a higher correlation with Step 1 score were weighted more heavily than those with lower correlations, and practice tests that were taken closer to actual test dates were weighted more heavily as well. After updating the predictor, 19 responses that were not used in creating lines of best fit for the predictor were used to test its accuracy (Table 3).

**Table 3:** Predicted USMLE Step 1 score vs. actual score USMLE: United States Medical Licensing Examination

Student	Predicted score	Actual score	Difference between predicted and actual scores
1	216.6	211	5.6
2	226.9	227	-0.1
3	243.2	230	13.2
4	240.7	234	6.7
5	239.4	237	2.3
6	241	237	4.0
7	239.4	243	-3.6
8	245.8	247	-1.3
9	239	247	-8.0
10	244.2	248	-3.8
11	252.5	250	2.5
12	249.2	250	-0.8
13	247.6	251	-3.4
14	253.4	254	-0.6
15	252.9	254	-1.1
16	253.6	254	-0.4
17	249.1	256	-6.9
18	258	256	2.0
19	256.1	262	-5.9

**Figure 3:**
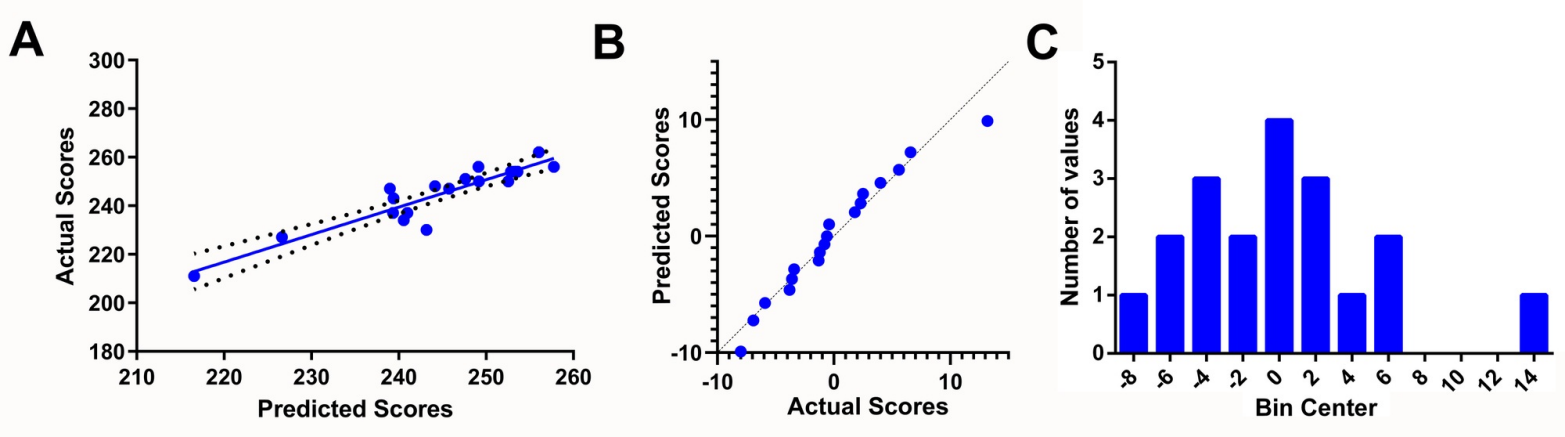
Correlation of predicted USMLE Step 1 scores with actual scores A: linear regression analysis of predicted scores using the score predictor and actual scores obtained on the USMLE Step 1 examination (R^2 ^= 0.845; slope = 1.131 ± 0.117; 95% confidence interval = 0.884 ± 1.379; n = 19); B: normal Q-Q plot indicating residuals were normally distributed; C: frequency distribution histogram of residual scores USMLE: United States Medical Licensing Examination

The linear regression of predicted Step 1 score outputs from the predictor to actual Step 1 scores showed a high correlation (Figure 3). Residuals were normally distributed.

## Discussion

Previous studies have indicated a significant but weak (17%) correlation between performance on the older version of MCAT and performance on USMLE Step examinations [12]. So far, only the University of Minnesota Medical School-Twin Cities has correlated scores on the new MCAT format with USMLE Step 1 scores (n = 220; multiple R = 0.44). This study, which sampled scores from students across the United States rather than a specific school (n = 282), corroborates their findings by finding a small but significant correlation between scores obtained on the new MCAT format and Step 1 performance (21%).

While enrolling in commercial coaching classes does not improve Step 1 performance, the importance of self-assessment practice questions and question banks in improving student understanding of the subject while simultaneously enhancing performance on standardized tests is well-known [23,24]. For example, students who self-assessed with 4,001-6,000 practice questions scored higher on USMLE Step 1 than those who took less than 2,000 questions [19]. Out of 399 usable responses in this study, 390 participants (97.7%) elected to use at least one practice test; 383 participants (96%) used UWorld QBank as a resource. While the number of practice tests varied between participants (mean: six tests; range: 0-14), there was a very clear association between the number of practice tests taken and Step 1 scores, with participants who took between six and nine practice examinations receiving the highest scores.

Arguably, the most fascinating result of this study, however, was the finding that UWSA2 scores might be best correlated with Step 1 scores. Considering that UWSA2 is produced by a company that is not directly involved with the development of Step 1 examinations, it was surprising to note that it more closely matched Step 1 scores as compared to self-assessment tests released by the NBME. While the sample size did have a slight effect on correlation, it was not the only factor. An alternative explanation could be the use of different grading curves on the NBME CBSSA tests as compared to the UWSA tests. It is possible that the UWSA2 grading curve more closely matches the grading curve on Step 1 examinations and, therefore, correlates better. However, correlating the average score of each selected practice test with the R^2^ value of its line of best fit revealed an R^2^ value of 0.256, suggesting that additional factors besides the grading curve contribute to the higher predictive value of UWSA2.

Another surprising finding of this study was the efficacy of the predictive model. A previous model used performance on CBSE and UWorld QBank, obtaining a grade of A during preclinical years, and receiving a need-based scholarship to predict Step 1 scores [17]. However, for this model to be viable, answers to all variables needed to be entered; any missing data would significantly hamper its ability to predict Step 1 scores. In contrast, data from our study showed that performance on practice tests are sufficient to predict Step 1 scores accurately. Additionally, the Step 1 score predictor used in our study allowed reporting but did not require the scores of the 14 different NBME CBSSA and UWorld practice assessments, UWorld QBank, and the NBME Free 120 questions. Given the high correlation between predicted and actual Step 1 scores, this score predictor becomes a useful tool to self-assess progress when studying for Step 1.

There were several limitations to this retrospective study. The study required students to volunteer and report their scores, and this may have resulted in a selection bias. Although the mean MCAT score of participants was similar to the national mean of matriculants who would appear for Step 1 examination in 2019, the mean Step 1 score was significantly higher than the national average. This indicated that the cohort of participants was significantly skewed towards higher-scoring students, possibly because students who score higher are more likely to volunteer to report their scores. We were also unaware of other preparatory materials the students may have used that could have a significant effect on their performance. Nevertheless, this study provides further evidence that correlates scores on the new MCAT format with Step 1 scores; It also delineates the contributions of specific self-assessment tests and question banks towards performance on Step 1 examinations and highlights their predictive value.

## Conclusions

This study utilized a cohort not restricted to a particular university to correlate scores on the new MCAT format with USMLE Step 1 scores as well as to analyze widely used self-assessment resources currently available for Step 1 preparation. Our results confirm the importance of MCAT not only as a stratifying factor for medical school admissions but also as a significant predictor of Step 1 outcomes. Importantly, data obtained from this study allowed an existing score predictor to be updated and validated. The results of this study will help cost-conscious students and undergraduate medical institutions to select appropriate self-assessment tests for evaluating their preparation for the USMLE Step 1 examination and remove the ambiguous interpretation of practice test scores with perceived progress. Additionally, the updated USMLE Step 1 score predictor will allow students to self-evaluate and gauge their progress in real-time. Given the ever-increasing competition in obtaining residency positions, it is hoped that the results of this research will help prepare and assess the readiness of students prior to taking the examination and thereby help reduce student stress and improve student wellness.

In February 2020, FSMB and NBME announced pass/fail reporting for Step 1 examinations held after January 2022 to decrease the emphasis on USMLE in medical schools. Due to the imminent loss of Step 1 as a screening tool for residency selection, residency directors will need to develop novel methods to select interviewees, such as a standardized video interview, to help bolster a holistic approach to resident selection. However, since Step 1 scores significantly correlate with student performance in medical clerkships, Step 2, and residency, adequate preparation for Step 1 needs to continue for achieving overall success in medical education.
